# The Role of the Mediterranean Diet in the Prevention of Sarcopenia and Frailty in Older Adults: A Narrative Review

**DOI:** 10.3390/nu17101743

**Published:** 2025-05-21

**Authors:** Marta Arroyo-Huidobro, Magali Amat, Aina Capdevila-Reniu, Ariana Chavez, Martina Pellicé, Andrea Ladino, Constanza Sepúlveda, Emilio Sacanella

**Affiliations:** 1 Geriatric Department, Hospital Clinic de Barcelona, University of Barcelona, Villarroel Street 170, 08036 Barcelona, Spain; kachavez@clinic.cat (A.C.); pellice@clinic.cat (M.P.); csepulveda@clinic.cat (C.S.); 2 Physiotherapy Department, Hospital Clinic de Barcelona, 08036 Barcelona, Spain; mamat1@clinic.cat; 3 Internal Medicine Department, Hospital de Mataró, Consorci Sanitari del Maresme, 08304 Mataró, Barcelona, Spain; acapdevilare@csdm.cat; 4 Internal Medicine Department, Hospital Clinic de Barcelona, 08036 Barcelona, Spain; ladino@clinic.cat; 5 Department of Medicine, Faculty of Medicine and Life Sciences, University of Barcelona, 08036 Barcelona, Spain; 6 Institut d’Investigacions Biomèdiques August Pi I Sunyer (IDIBAPS), 08036 Barcelona, Spain; 7 Centro de Investigación Biomédica en Red, Fisiopatología de la Obesidad y la Nutrición (CIBEROBN), Instituto de Salud Carlos III, 28029 Madrid, Spain

**Keywords:** sarcopenia, Mediterranean diet, frailty, function, nutritional assessment, elderly

## Abstract

**Background/Objectives**: Sarcopenia and frailty are interrelated conditions and have a high incidence in older adults. They contribute to increased morbidity and mortality and poor quality of life. There is emerging evidence that healthy diets such as the Mediterranean diet could delay the onset of sarcopenia and frailty. This review aims to evaluate the role of the MD in preventing these conditions. **Methods**: A literature search was conducted on PubMed (MEDLINE, NCBI) for English-language articles published within the last 10 years (2014–2024) using the search terms “Mediterranean diet”, “frailty”, “sarcopenia”, and “old people”. A total of 111 articles were identified, of which 36 were excluded during the initial screening. Subsequently, 75 manuscripts were assessed for eligibility. Subsequently, a further 62 articles were excluded (narrative reviews, articles not focused on the elderly population, or articles with different outcomes). Finally, 13 articles were included in the review. **Results**: The 13 selected studies comprised seven observational studies, three systematic reviews and meta-analyses, and three clinical trials. The findings suggest that adherence to the Mediterranean diet (MD), particularly when combined with physical activity, may improve body composition and cardiometabolic health and reduce indicators of sarcopenia in obese older adults. Furthermore, MD-based nutritional interventions were associated with improved physical functions such as balance, gait, fall risk, flexibility, and muscle strength (*p* < 0.05, all). The MD also demonstrated a preventive effect against frailty, particularly in pre-frail individuals. **Conclusions:** High adherence to the Mediterranean diet (MD) may delay the onset of sarcopenia and improve muscle function in older adults. However, the available scientific evidence is of low to moderate quality. Well-designed prospective intervention studies are needed to confirm whether the MD can modify the natural history of sarcopenia and/or frailty in older adults.

## 1. Introduction

Frailty and sarcopenia are common health problems that significantly impact the quality of life of older adults. Frailty is characterized by decreased functional reserve and increased vulnerability to stressors, which can lead to adverse health outcomes such as falls, hospitalization, and mortality [1,2,3]. Sarcopenia, which is defined as the loss of skeletal muscle mass and function, is a major component of frailty and is associated with similar adverse outcomes [4,5]. The prevalence of frailty among older people ranges from 4% to 59%, depending on the population and the criteria used. Sarcopenia affects approximately 5% to 10% of the general elderly population, with higher rates observed in individuals with chronic diseases [6,7]. Both conditions are particularly prevalent in patients with heart failure, cancer, and dementia, with rates reaching 75% and 47%, respectively [8]. Frailty and sarcopenia are strong predictors of morbidity and mortality, and they also increase the risk of adverse outcomes (e.g., falls, fractures, and institutionalization), which is even higher in patients suffering from both conditions. This leads to higher healthcare utilization and poorer quality of life [3,9,10,11,12,13,14].

Malnutrition is a common geriatric syndrome that is often overlooked among the elderly, affecting up to 30–50% of older adults in hospitals and long-term care facilities. Age-related physiological changes, chronic diseases, and social factors significantly contribute to nutritional decline. It is associated with an impaired immune response, higher rates of hospitalization, and morbidity and mortality [3,12] and also emerges as a critical factor in the development of sarcopenia and/or frailty [3,12,13,14]. Thus, inadequate nutrient intake can precipitate or exacerbate both processes; however, it seems that a balanced diet and/or nutritional supplements, such as protein, vitamin D, and omega-3 fatty acids, combined with physical activity (e.g., resistance training), have been shown to improve muscle mass and function [5,11]. It is currently well established that the most effective nutritional intervention is achieved through healthy dietary patterns that provide a wide variety of nutrients, rather than through the isolated prescription of vitamins, nutrients, or trace elements. One of the healthiest and most complete dietary patterns is the Mediterranean diet, whose numerous beneficial effects in the prevention of various diseases are well known. However, there is limited information on its potential effect in preventing sarcopenia and frailty in older adults [13,14,15].

**Figure 1:**
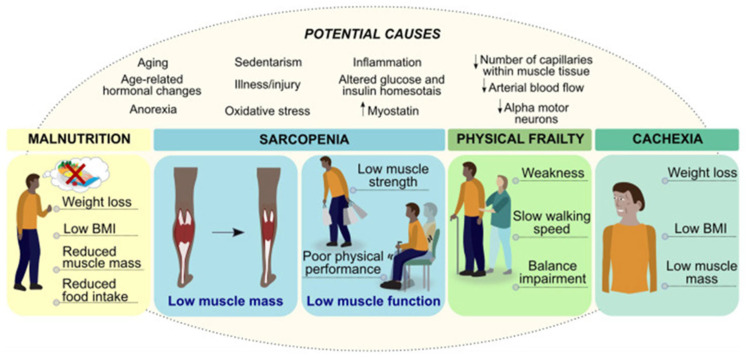
Interactions between malnutrition, sarcopenia, physical frailty, and cachexia [7].

The Mediterranean diet (MD) is characterized by a high intake of plant-based foods, olive oil as the main source of fat, and moderate consumption of seafood, dairy products, poultry, and eggs. Wine is consumed in moderation, and red and processed meats and sugary foods are consumed in limited quantities [16]. Most of these foods are rich in antioxidants and anti-inflammatory molecules, as well as omega-3 fatty acids, which are believed to be responsible for the well-known beneficial effects of the Mediterranean diet [17]. In this sense, the MD has been shown to protect against cardiovascular disease, improve blood pressure control and lipid profiles, and enhance endothelial function [18,19,20]. Likewise, this diet can reduce the risk of type 2 diabetes, obesity, and metabolic syndrome [21,22]. Some authors have even suggested that high adherence to the MD could reduce the risk of cancer and delay cognitive decline [1,23,24]. More recently, research studies have proposed that this dietary pattern can improve physical function and mobility, reduce the risk of frailty, and promote better overall health [13,14,15] The objective of this review is to analyze the scientific evidence regarding the potential protective role of the Mediterranean diet against the development of sarcopenia and frailty and its ability to enhance physical function in older people. We also aim to assess whether the positive effects of this dietary pattern can be generalized to all elderly people (non-frail, pre-frail, and frail subjects) (see Figure 1).

## 2. Materials and Methods

### 2.1. Search Strategy, Selection Criteria, and Data Extraction

**Figure 2:**
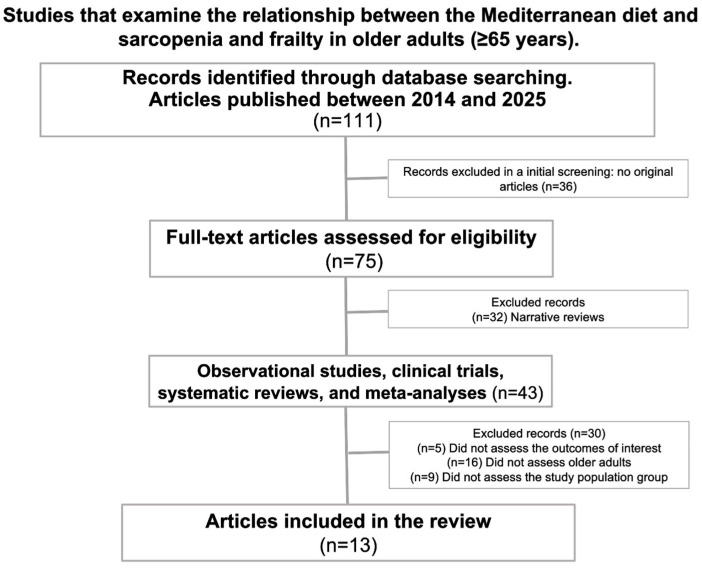
Flow chart of the selection process.

A comprehensive search of the scientific literature published in PubMed was conducted to identify articles on the relationship between adherence to the Mediterranean diet (MedDiet), sarcopenia, and frailty. The literature search was conducted on 12 October 2024 (initial search) and 15 January 2025 (final search) using the Medline database (PubMed interface). The PubMed search strategy included the following keywords and combinations: (“Mediterranean Diet” OR “MeDiet”) AND (“Sarcopenia”) AND (“Frailty”). The selection of studies was conducted in two phases. First, titles and abstracts were reviewed to determine their relevance based on the established criteria. Secondly, a full-text review was carried out in which the selected articles were retrieved and analyzed in depth to evaluate their methodology, results, and conclusions. A total of 111 articles were identified, of which 36 were excluded in an initial screening, leaving 75 manuscripts to be assessed for eligibility. A further 32 narrative reviews were excluded, as were another 30 manuscripts that did not focus on the elderly population or specifically assess the relationship between the Mediterranean diet and sarcopenia, frailty, or physical function. Ultimately, only 13 studies focusing on elderly populations and assessing the relationship between adherence to the Mediterranean diet and sarcopenia, frailty, or physical functionality were included in the review. A flow chart showing the selection process is presented in Figure 2.

### 2.2. Eligibility Criteria

Studies examining the relationship between the Mediterranean diet and functionality in older adults (aged 65 years or over) published between 2014 and 2024 were included. This includes research investigating the impact of adherence to the Mediterranean diet on physical, cognitive, or social functioning in this age group. Only studies published in English or Spanish will be considered. Eligible studies must be observational studies, clinical trials, systematic reviews, or meta-analyses that provide comprehensive data on the topic.

However, studies that do not specifically focus on older adults (aged 65 years or over) were excluded. Studies that do not directly address the Mediterranean diet or use non-standard definitions of the diet were not included. Additionally, publications that are not available in full text were excluded from the review. Narrative review studies were also excluded.

### 2.3. Data Analysis

The data extracted from the selected studies were organized into a synthesis matrix including the following:

- Author, year of publication, and place.
- Study group
- Study design
- Outcome definition
- Sample size
- Mean age
- Results
- Controlled variables

The findings will be summarized both qualitatively and quantitatively, highlighting the observed trends, implications for practice, and recommendations for future research. The nomenclature and terminology of the study variables will be presented.

### 2.4. Mediterranean Diet Scores

The Mediterranean diet can be assessed using various scales or scores. The most common method of evaluating adherence to the Mediterranean diet is the Mediterranean Diet Score (MDS), which is a 10-point scale. Each of the nine specified components is assigned a score of 0 or 1. For beneficial components such as vegetables, legumes, fruits, nuts, cereals, and fish, individuals whose consumption falls below the median receive a score of 0, while those whose consumption meets or exceeds the median receive a score of 1. The total score ranges from 0 to 9, with higher scores indicating greater adherence to the Mediterranean diet [25].

We have also included studies that use the modified V-Med diet and the alternate Mediterranean diet score (aMED) [26], which assigns points to nine components for a total of 9 points. Intake of these nine food groups was dichotomized using sex-specific median values as cut-off points. A score of 1 was given for consumption above the median for beneficial foods (whole grains, vegetables, fruits, nuts, legumes, fish, and the monounsaturated-to-saturated fat ratio), and below the median for detrimental foods (red and processed meat), while a score of 0 was given for everything else.

Other tests used include the validated 14-item questionnaire from the PREDIMED study being 14 points, a maximum score [27]. Briefly, each item was assigned a score of 1 or 0 (0–5 indicating the lowest adherence, 6–8 indicating average adherence, and 9 or more indicating the highest adherence). Another test used was the 14-item Mediterranean Diet Adherence Screener (MEDAS), in which each item was scored as 0 or 1, yielding a maximum score of 14. A score of ≥10 suggests high adherence, a score of 6–9 suggests moderate adherence, and a score of ≤5 suggests low adherence. Finally, a modified version of the MEDI-LITE was used, where a score of 12 or greater indicated high adherence, a score between 9 and 11 represented moderate adherence, and a score of 8 or lower denoted low adherence [24].

### 2.5. Other Dietary Assessment Scores:

The dietary assessment was conducted to quantify food and beverage intake with known portion sizes and to ensure accurate record-keeping through interviews. Additionally, dietary data, including beverage intake, were collected through seven-day food diaries. One study [28] used a Food Frequency Questionnaire (FFQ), which is a semi-quantitative questionnaire based on 126 food items with nine frequency options ranging from “never or less than once a month” to “six or more times a day” [29,30]. Another study used a 217-item FFQ, categorizing adherence levels as follows: 0–3 (low adherence), 4–6 (moderate adherence), and 7–10 (high adherence) [31]. Another study was conducted using a validated 143-item semi-quantitative food frequency questionnaire [32], or a photographic food atlas containing over 1000 photographs of known portion sizes was used during a face-to-face interview between the patient and a qualified nutritionist [33].

The Korean Healthy Eating Index (KHEI) was also used to quantify adherence to dietary guidelines for Korean adults. According to an eating guide developed by the National Heart, Lung, and Blood Institute, the Dietary Approaches to Stop Hypertension (DASH) scores eight components (seven food groups and one nutrient)—each worth 5 points—for a maximum total of 40 points. Finally, food records were collected by a registered nutritionist [34].

### 2.6. Definitions of Sarcopenia, Physical Performance, and Frailty

Regarding sarcopenia, the European consensus definition (EWGSOP and EWGSOP2) was applied, wherein sarcopenia was assessed using three parameters: (a) low muscle strength, (b) low skeletal muscle mass, and (c) reduced physical performance. One article used sarcopenia indexes measured by dual-energy X-ray absorptiometry (DEXA) [32], while another used dynapenia and low appendicular skeletal muscle mass [24].

Physical performance was also evaluated using various methods. Hand grip strength (HGS) was measured by quantifying the amount of static force exerted by the hand and forearm around a hand-held dynamometer. Low HGS is defined as a value of 28.6 kg for men and 16.5 kg for women, or a value of <20 kg for women and <30 kg for men [34,35]. Other methods include the Timed Get-Up-and-Go test (scoring < 14 s as independent mobility, 15–20 s as semi-independent mobility, and 20–30 s as dependent mobility), the 6 m walk test (normal range: 400–700 m), the 10 m walk test, and the Berg Balance Scale (BBS) (scores below 36 indicate an increased risk of falls, scores between 37 and 45 indicate the need for a walking aid to walk safely, and scores above 45 indicate no increased risk of falls). The Four Square Step Test (FSST) and the 30 s chair stand test are also used [28]. Another measure is gait speed (a risk of sarcopenia is defined as <0.8 m/s) [36], and the Tinetti scale evaluates balance, gait, and fall risk [37]. Finally, the Short Physical Performance Battery (SPPB) test evaluates lower extremity function by measuring three domains of physical function that mimic activities of daily living: (1) balance, (2) gait speed, and (3) lower extremity strength. The scores from each test are ranked using a 0–4 scale.

Frailty was assessed using the frailty phenotype [38], the Frail Scale, or the Fried assessment [39,40]. Disability was evaluated using different methods and definitions: Activities of Daily Living (ADLs), the Instrumental Activities of Daily Living (IADLs) scale, and the Physical Functioning Questionnaire.

## 3. Results

### 3.1. Characteristics of Included Studies

Of the 111 articles initially identified as analyzing the protective role of the Mediterranean diet in preventing sarcopenia, loss of functional autonomy, and frailty, 13 manuscripts were ultimately included in the review (Figure 2). These manuscripts focused on populations over 65 years old. Of these, three were meta-analyses (total subjects = 60,252) [12,38,41], seven were cross-sectional studies (total subjects = 26,230) [24,28,31,32,34,35,42], and three were clinical trials (total subjects = 764) [36,37,43]. A total of 87,884 older adults (aged 65–86) from Spain, Italy, Korea, Israel, Brazil, and other European countries were included in the study. Most of the studies were conducted in Mediterranean countries and focused on community-dwelling individuals [41]. In most studies, women represented the largest portion of the sample, with the reported mean age ranging from 65 to 86 years. The main characteristics of the studies are summarized in Table 1.

#### 3.1.1. The Mediterranean Diet and Sarcopenia

The following studies [32,34,41,42,43] suggest that combining the Mediterranean diet (MD) with exercise can lead to significant improvements in body composition and cardiometabolic health in older adults, as is described later. Interventions such as water-based resistance training and a caloric-restricted MD have demonstrated positive effects on muscle mass and fat reduction; however, their impact on body image is unclear. Although a higher-quality diet is associated with improved handgrip strength, the effects on sarcopenia and muscle strength markers are inconsistent. These results indicate that, although diet and exercise contribute to physical health, more individualized approaches may be needed to address sarcopenia and related conditions in the elderly.

A study by Martínez-Rodríguez et al. [43] assessed the effects of water-based resistance training on body composition, body image perception, and adherence to the MD in older women participating in a nutrition education program for 14 weeks. The study involved 34 participants, who were divided into two groups: One group participated in water-based resistance training combined with a nutritional intervention based on the MD (the interventional group), while the control group received only the nutritional intervention based on the MD. No significant differences were found between the groups regarding body image perception or adherence to the MD. However, the interventional group showed a significant increase in muscle mass (2 ± 5.85 kg, *p* < 0.001) and a decrease in fat mass (*p* < 0.001) compared to the control group. This suggests that, although water-based resistance training did not affect body image or adherence to the MD, it led to significant improvements in body composition.

The PREDIMED-Plus study [32] applied a multicomponent intervention to a cohort of older adults with metabolic syndrome. The intervention group (IG) was subjected to a hypocaloric Mediterranean dietary intervention (including olive oil and nuts) together with physical activity promotion (at least 150 min of moderate-to-vigorous exercise per week) and behavioral support (including dietary counselling, goal setting, and self-monitoring). The control group (CG) followed a non-caloric-restricted Mediterranean diet and received general health advice. The intervention group experienced significant weight loss and improvements in body composition and cardiometabolic health. After one year, the IG had lost an average of 3.2 kg, compared to 0.7 kg in the CG. Thirty-three percentage of IG participants achieved ≥ 5% weight loss (vs. 11.9% in the CG, *p* < 0.001). Significant reductions were observed in waist circumference, fasting glucose, triglycerides, and inflammatory markers, while HDL cholesterol and insulin sensitivity improved (*p* < 0.05). After three years, IG participants showed further reductions in total fat mass (−0.38%) and visceral fat (−70.4 g), as well as better lean mass retention (+0.34%) (*p* < 0.001). The PREDIMED-Plus study suggests that a multicomponent intervention comprising an energy-restricted Mediterranean diet (MD), physical activity, and behavioral support can effectively enhance body composition and metabolic health, thereby counteracting age-related fat gain and muscle loss in individuals with metabolic syndrome. Adherence to the intervention was high. However, some limitations of the study must be taken into account when interpreting the results. For example, as this was a cross-sectional study, no causal relationships can be inferred. Furthermore, the definition of sarcopenia may under- or overestimate its prevalence, and finally, self-reported dietary questionnaires may introduce bias.

In contrast, a cross-sectional study by Stanton et al. [42] investigated the association between adherence to the MD and sarcopenia in obese older people. Surprisingly, higher adherence to the MD was not associated with a significant improvement in sarcopenia markers such as handgrip strength (HGS) or the Short Physical Performance Battery (SPPB). None of the participants were diagnosed with sarcopenia or sarcopenic obesity at the time of recruitment, leading the authors to suggest that in obese older adults, factors such as muscle strength or gait performance may not necessarily correspond with muscle mass loss. The main limitations of this study were that it was a cross-sectional study, the sample included only obese older adults, and the physical activity of the participants was not assessed.

Kim H et al. [34] conducted a cross-sectional study in Korea in 3675 elderly subjects (71–73 years old), examining the relationship between diet quality and hand grip strength using three indices: the Korean Healthy Eating Index (KHEI), the Alternate Mediterranean Diet (aMED), and the Dietary Approaches to Stop Hypertension (DASH) using data from the Korean National Health and Nutrition Examination Survey. The authors concluded that higher diet quality, in each of the indices used, was associated with a 32–53% lower risk of low hand grip strength in older Koreans. The results suggest that higher diet quality, as indicated by better punctuation on these scores, was strongly associated with lower odds of experiencing low HGS, which is suggested as a marker of pre-sarcopenia. The main limitations of this study were the following: It was a cross-sectional study, and the subjects included were not originally from Mediterranean countries.

In another cross-sectional study based on baseline data from the PREDIMED-Plus study, Abete et al. [32] included 1535 obese or pre-obese subjects with metabolic syndrome and categorized them according to sex-specific tertiles of the sarcopenic index (SI) as assessed by dual-energy X-ray absorptiometry. Multiple adjusted linear regression models showed significant positive associations across tertiles of SI with adherence to the Mediterranean diet (*p* trend < 0.05), physical activity (*p* trend < 0.0001), and the 30 s chair-stand test (*p* trend < 0.0001). Specifically, participants in the highest tertile of the Mediterranean diet score had a significantly lower likelihood of sarcopenic obesity compared to those in the lowest tertile. In addition, increased physical activity was inversely associated with the risk of sarcopenic obesity. These findings suggest that improved adherence to the Mediterranean diet and regular physical activity are key factors in slowing the sarcopenic process and ensuring healthy ageing. Some limitations of this study should be mentioned: A cross-sectional design does not allow one to suggest causal effects; the specific definition of sarcopenic obesity may underestimate/overestimate the incidence of sarcopenia; and finally, dietary and physical activity data come from self-reported questionnaires, so this information could be biased.

Finally, another observational study by Coelho-Junior [24] in a sample of 491 Italian subjects over 65 years of age investigated whether adherence to DM and regular aerobic physical activity reduced the risk of sarcopenia. Eighty-eight percent of the subjects had moderate to high adherence to the DM, and the incidence of sarcopenia was low (5.3%). There were no significant differences in sarcopenia between the low, moderate, and high adherence groups. Although the results were negative, the limitations of the study should be highlighted: It was an observational cross-sectional study; the information collected was not complete; and the MED-LITE scale for measuring adherence to the diet is not optimal.

#### 3.1.2. Mediterranean Diet and Functionality

Carcelén-Fraile et al. [37] included 116 older adults in a 12-week clinical trial. The intervention group (n = 57) received a program of yoga plus Mediterranean diet, while the control group (n = 59) received no intervention. The yoga intervention (times/week) was designed to address various aspects of physical fitness such as strength, flexibility, balance, and stability. The MD intervention consisted of a dietary protocol with a meal plan that included the consumption of key foods in this dietary pattern (virgin olive oil, cereals, fruits, vegetables, and legumes) and the restriction of other foods such as red meat, cheese, processed meat, butter, and sugary drinks. The intervention had a positive effect on nutritional status, balance, gait, fall risk, flexibility, and muscle strength. The 12-week intervention combining yoga and the Mediterranean diet led to significant improvements in flexibility (Cohen’s d = 0.37–0.64) and strength (Cohen’s d = 0.39–0.81) in older participants. These benefits were seen in both upper and lower body strength and flexibility measures. There were improvements in body weight and body mass index, lipid profile, blood pressure, and inflammatory markers. The strengths of the manuscript were its design as a randomized controlled trial, good follow-up of participants, and high adherence to the intervention. On the other hand, there were some limitations, such as the relatively small sample size, the short duration of the intervention, and the difficulty in separating the net effect of yoga and the MD intervention on the observed outcomes.

In 2018, Silva et al. [38] conducted a systematic review and meta-analysis examining the association between adherence to the Mediterranean diet and musculoskeletal and functional outcomes in community-dwelling older adults. The analysis included 12 studies with a total of 20,518 participants. The results showed that higher adherence to the Mediterranean diet was inversely associated with frailty, with an odds ratio (OR) of 0.42 (95% confidence interval [CI]: 0.28–0.65), and with functional disability, with an OR of 0.75 (95% CI: 0.61–0.93). These results suggest that individuals with higher adherence to the Mediterranean diet had 58% lower odds of frailty and 25% lower odds of functional disability compared with those with lower adherence. A meta-analysis for sarcopenia was not performed because of the considerable variability among the included studies. Cohort data showed no association between adherence to the Mediterranean diet and sarcopenia, but cross-sectional results showed a positive association. However, this meta-analysis also has some limitations (methodology, only baseline data of patients, high heterogeneity, different criteria for frailty and functional disability), and its results should be interpreted with caution.

In the same order, the study by Barrea et al. [35] investigated the association between adherence to the Mediterranean diet, as assessed by the PREDIMED questionnaire, and hand grip strength (HGS) in older women. The observational study included 84 women aged 60–85 years. The authors found that women with higher adherence to the Mediterranean diet had better HGS. Specifically, 39% of participants with an HGS above the 20 kg threshold showed high adherence to the diet, compared with only 14% of those with lower HGS values (*p* = 0.018). After adjusting for body mass index, lower adherence to the Mediterranean diet was associated with reduced odds of achieving a higher HGS (*p* < 0.001). These results suggest that adherence to the Mediterranean diet is positively associated with muscle strength in active older women. Some limitations of the study include its cross-sectional design, sample size, and the cut-off points of HGS for obese or non-obese subjects, and its results should be interpreted with caution.

In another cross-sectional study involving 117 patients, Tepper et al. [28] examined the relationship between adherence to the Mediterranean diet and physical function in older adults with type 2 diabetes. The researchers hypothesized that adherence to the Mediterranean diet would be associated with better physical function (6 and 10 min walk test, timed get up and go, 30 s chair-stand, grip strength, and instrumental activities of daily living) in this population and that the association might vary by age. The study found that higher adherence to the MD was associated with better physical function indices, such as improved mobility and strength. However, the strength of this association varied by age group, suggesting that age may play a role in how the MD affects physical function in older adults with type 2 diabetes. These findings suggest that following a Mediterranean diet may contribute to better physical function in older adults with type 2 diabetes, although the benefits may vary by age. Again, the main limitations of the study were its design as a cross-sectional study, the sample size, and the selected population included (older diabetic subjects with high educational level), which makes it difficult to generalize to other patients.

Coelho-Junior et al. [41] conducted a systematic review and meta-analysis of cross-sectional and longitudinal studies. The primary or secondary outcome was the association of adherence to MD with physical performance and/or cognitive function in non-demented older adults. Nineteen cross-sectional studies included 19,734 community-dwelling and institutionalized older adults without disability or dementia. High adherence to MD was associated with better walking speed, knee muscle strength speed, and global cognition (*p* < 0.01, all). However, the meta-analysis of 34 longitudinal studies including 98,315 community-dwelling people with a mean follow-up of 3–12.6 years also showed better global cognition in patients with high adherence to MD, but there was no significant improvement in subjects’ mobility problems. Although important, the results should be interpreted with caution due to heterogeneity and publication bias.

#### 3.1.3. Mediterranean Diet and Frailty

A meta-analysis published by Domínguez et al. [12], including 11 cohort studies with a total of 106,615 people over 60 years of age followed for 1 year, analyzed the relationship between adherence to the MD and the incidence of frailty. The diagnosis of frailty was based on Fried’s criteria. The authors observed a 45% reduction in the incidence of frailty in patients with high adherence to MD compared with those with low adherence and suggested that there is an inversely proportional relationship between adherence to MD and the incidence of frailty. According to the authors, the anti-inflammatory effect of several key components of the diet (vegetables, extra virgin olive oil, and fruits) could be responsible for this beneficial effect and lend plausibility to the association found.

In a large study by Zhang et al. [31] of 21,643 hospitalized women from the United Kingdom Women’s Cohort Study (UKWCS) over 13 years, 14,838 (68.6%) were identified as frail using the hospital frailty risk score. In this cohort, women with high adherence to the Mediterranean diet had a lower risk of frailty (HR = 0.89, 95% CI: 0.85–0.94), while moderate adherence also showed a modest protective effect (HR = 0.95, 95% CI: 0.91–0.99). Subgroup analyses showed that while high adherence remained protective in all groups, the protective effect of moderate adherence was less pronounced in women aged ≥ 60 years and those with a BMI > 24.9 kg/m^2^. The main limitations of the study were its cross-sectional design, the recruitment of patients, and data by postal questionnaire, and a high proportion of subjects included were younger than 65 years, and 100% of them were women.

Finally, the study by Shankar Ghosh [36] examined the effect of a one-year intervention with MD on the microbiome, which is associated with a lower risk of frailty in a cohort of European community-dwelling elders aged 65–79 years (n = 612). The authors conclude that this dietary pattern can modify the gut microbiome, which in turn has the potential to promote healthier aging and reduce frailty. The main limitation of the study is that it analyzes the changes that the Mediterranean diet can induce in the microbiota and that are associated with a lower risk of frailty, but it does not directly assess clinical parameters of frailty (muscle strength, mobility, or sarcopenia).

## 4. Discussion

This narrative review examines the relationship between adherence to the Mediterranean diet (MD) and sarcopenia, frailty, and functional status in older adults. The findings suggest that older people who adhere to the MD and engage in regular physical activity are less likely to develop sarcopenia or functional disability. Specifically, MD-based nutritional interventions were associated with improvements in several physical functions, such as balance, gait, fall risk, flexibility, and muscle strength (*p* < 0.05 for all). These results are consistent with the main findings of a meta-analysis and systematic review, which reported a 38% reduction in frailty risk in individuals with moderate adherence to the MD, as assessed using the FRAIL scale or the modified Cardiovascular Health Study Frailty Criteria (mCHS) [38]. These beneficial effects of the MD and exercise appear to be more pronounced in pre-frail subjects than in frail ones. However, the scientific evidence available on this issue so far is of low to moderate quality.

Most of the studies included in this review are cross-sectional studies with small sample sizes [24,28,31,32,34,35,42], except for two Korean studies. The cross-sectional design makes it difficult to establish a causal relationship between adherence to the Mediterranean diet (MD) and a lower incidence of frailty or sarcopenia. Furthermore, these researchers use different criteria to define frailty and sarcopenia and to assess adherence to the MD. Physical function is also evaluated using different parameters (hand grip strength, walking speed, and the chair-stand test), and the enrolled population is heterogeneous. This variability makes it difficult to draw conclusions that can be applied to older people. There are only three clinical trials [36,37,43] involving 764 patients. However, 612 of these subjects were included in a study that did not directly evaluate the relationship between adherence to the Mediterranean diet and sarcopenia, frailty, or physical function in the elderly. Rather, it investigated whether this dietary pattern promotes changes in the microbiota that delay the onset of sarcopenia or frailty in the elderly [36]. The other two clinical trials [37,43] involve an intervention with DM and different types of physical activity, analyzing the effect on muscle mass or muscle function parameters. Although the results are positive in both studies, the sample sizes are small, the interventions are short-term, and it is not possible to determine long-term maintenance or loss. Finally, a meta-analysis by Coelho-Junior et al. [41] observed that, in 19,734 patients with high adherence to the Mediterranean diet, cognitive decline and walking speed were lower compared to those with low adherence. In another meta-analysis, Dominguez et al. [12] showed that high adherence to the MD is associated with a reduced risk of incident frailty in 103,615 subjects. However, the data from both meta-analyses come from observational studies with low to moderate quality of evidence.

Adherence to the Mediterranean diet (MD) has been associated with a lower risk of several non-communicable chronic diseases, including cardiovascular disease, cancer, cognitive decline, and fractures [17,18,19,20,21,22,23,24]. This healthy dietary pattern has also been identified as a key factor in the prevention of age-related chronic diseases and has been linked to a reduced risk of all-cause mortality. The main components of the MD (fruits, vegetables, olive oil, fish, and nuts) are rich in antioxidants and anti-inflammatory molecules and are an important source of polyunsaturated fatty acids. These have a proven capacity to reduce oxidative stress and the inflammatory response in various tissues and are believed to be responsible for the beneficial effects of the Mediterranean diet, including those related to aging, such as sarcopenia [17].

Sarcopenia is an age-related condition characterized by the progressive loss of skeletal muscle mass and strength. Poor nutrition and low physical activity play a crucial role in its development [3,12], and it has been identified as a precursor of frailty and functional decline in older people. Currently, there is no pharmacological treatment for sarcopenia, and the main therapeutic options for preventing or delaying its onset are to follow a healthy diet and remain physically active in old age [44]. Indeed, most nutritional intervention studies suggest that dietary patterns are more important than the intake of specific nutrients or micronutrients (e.g., vitamins and trace elements) for achieving positive outcomes [11,35,43]. Physical activity is another key component of a healthy lifestyle, and resistance training is the most effective way for older adults to prevent a rapid decline in muscle strength and mass [32].

Although most of the reviewed studies suggest that a Mediterranean diet and physical activity have a beneficial effect on the prevention of sarcopenia and frailty, there are some limitations that require the results to be interpreted with caution, as bias could have been introduced. Firstly, considerable heterogeneity was observed among the included studies; most of these were cross-sectional, making it difficult to establish causal relationships. Secondly, the study populations were characterized by overrepresentation of women and a quite variable age range (60–86 years old), so the results for men or for different age groups could be different. Thirdly, different scores were used to measure adherence to the Mediterranean diet (aMED, PREDIMED questionnaire, MDS, MEDAS, and MEDI-LITE), which may affect comparability between studies. Fourthly, there is potential for recall bias when self-reported questionnaires are used to evaluate adherence to the Mediterranean diet or to assess daily nutritional intake, as these may over- or underestimate adherence to the MD and nutritional intake. Physical activity was not consistently or adequately reported throughout the studies. Sixthly, the definition of frailty and the diagnostic criteria for sarcopenia were not consistent across all studies. Finally, many of the interventions were multicomponent, making it challenging to isolate the specific effects attributable to the Mediterranean diet itself.

In summary, some studies suggest that the Mediterranean diet, especially when combined with physical activity, could be an effective way of preventing or delaying sarcopenia and frailty in older people. However, the quality of the current evidence is low, so well-designed longitudinal interventional studies focusing on older people are needed to confirm these preliminary associations. If these findings were confirmed by intervention studies, a non-pharmacological, multicomponent therapy comprising the Mediterranean diet and exercise could be used to delay sarcopenia and improve muscle function in older people. This would be the most effective way to promote healthy aging and delay the onset of frailty in later life.

## 5. Conclusions

The Mediterranean diet (MD) is a highly effective, multidimensional nutritional approach that promotes functional health and prevents age-related decline. However, there is no consistent evidence regarding the MD’s role in preventing sarcopenia and frailty in older people. Future research answering this question should rely on long-term, multicomponent intervention studies combining the MD with physical activity in different cohorts of older, pre-frail adults. If the preliminary findings are confirmed, our aging society may benefit.

## Figures and Tables

**Table 1:** Main data of the studies included in the review.

Author, Publication Year, and Place	Study Group	Study Design	Outcome Definition	Sample Size	Mean Age (Years)	Results	Controlled Variables
OBSERVATIONAL STUDIES							
Tepper et al., 2018, Israel [28]	Community dwellers	Cross-sectional study	Association between adherence to Mediterranean diet (FFQ) and physical function indices	117	70.6 ± 6.5	6 min walk (*p* = 0.001), and 10 min walk (*p* = 0.02), berg balance (*p* < 0.001)	Physical assessment (Berg Balance test, Timed Get-Up-and-Go, 6 min walk, 10 m walk, Four Square Step Test, 30 s chair stand, and Grip strength), and IADL
Kim, H., 2019, Korea [34]	Community dwellers	Cross-sectional study	Association between HGS and three indices—the Korean Healthy Eating Index (KHEI), the Alternate Mediterranean Diet (aMED), and Dietary Approaches to Stop Hypertension (DASH)	3675	71–73 years (men) and 71–74 years (women)	Risk of low HGS: Men: aMED (OR: 0.64, 95% CI: 0.44–0.93, *p*-trend = 0.014) Women: aMED (OR: 0.47, 95% CI: 0.31–0.69, *p*-trend < 0.001)	HGS, KHEI, Mediterranean diet, DASH diet
Abete, I., 2019, Spain [32]	Community dwellers	Cross-sectional analysis	Analyze the association of lifestyle variables (Mediterranean dietary score) with PA and sarcopenia	155	65.2 ± 4.9 years	PA (*p* < 0.0001), 30 s chair stand test (*p* < 0.0001).	Sociodemographic variables, sarcopenic indexes, anthropometric, clinical, and biochemical variables, and dietary variables
Barrea, L., 2019, Italy [35]	Community-dwelling women	Cross-sectional observational study	Association between MeDiet adherence and hand grip strength	84	71.7 ± 5.5 years	HGS was positively correlated with a PREDIMED score, OR 1.59.	Anthropometric measurements, adherence to the MD, and dietary assessment
Stanton, A., 2019, Australia [42]	Obese older adults	Cross-sectional analysis	Med diet adherence and whole body and regional body composition, grip-strength, SPPB	65	68.7 ± 5.6 years	Greater adherence was not associated with a decreased risk of sarcopenic symptomology (SPPB: OR = 0.51; 95% CI: 0.10–2.80; *p* = 0.438; muscle strength: OR = 1.81; 95% CI: 0.44–7.52; *p* = 0.409; gait speed: OR = 0.70; 95% CI: 0.24–2.0; *p* = 0.506)	Adherence med diet
Coelho-Junior, H., 2023, Italy [24]	Community dwellers	Cross-sectional observational study	Combined aerobic training and Mediterranean diet with a lower prevalence of sarcopenia	491	72.7 ± 5.7 years	No significant associations	BMI, walking activity, med diet, hand grip
Huifeng Zhang et al., 2023, Korea [31]	Community-dwelling women	Cohort prospective study	Evaluate the association between adherence to the Mediterranean diet and the risk of frailty	21,643	Women aged 65 years and older	High adherence had an 11% lower risk of frailty (HR = 0.89), while those with moderate adherence had a 5% lower risk (HR = 0.95)	Age, body mass index, physical activity levels, chronic diseases, socioeconomic status, smoking and alcohol, and medication use
CLINICAL TRIALS							
Martinez-Rodriguez, A., 2021, Spain [43]	Community-dwelling women	Randomized clinical trial Duration of 14 weeks	Evaluate how aquatic resistance interval training can influence body composition, body image perception, and adherence to the MeDiet	34	65 years	Muscle mass increased (*p* < 0.001)	Body composition (anthropometric measures), body image (Body Shape Questionnaire), and adherence to the MD
Carcelén-Fraile, M.D.C., 2024, Spain [37]	Community dwellers	Randomized controlled trial Duration of 12 weeks	Combined effects of yoga and the Mediterranean diet on various health outcomes in community-dwelling older adults	118	70.03 ± 2.58 years	Marcha d de Cohen = 0.41 (minor effect)	Nutritional status, flexibility, strength, balance, and fall risk
Shankar Ghosh, 2024, Europe [36]	Community dwellers	Randomized controlled trial Duration of 1 year	Mediterranean diet changes gut microbiome related to frailty	612	65–79 years	Healthy microbiome Lower inflammation markers	Diet, frailty status at baseline
SYSTEMATIC REVIEW AND META-ANALYSIS							
Silva, R., 2018, Brazil [38]	Community dwellers	Systematic review and meta-analysis	Association between adherence to a Mediterranean diet (MDS) and frailty, functional disability, and sarcopenia	20,518	68–84 years	N/S no pooled OR due to study heterogeneity	MDS, frailty phenotype, ADLS, IADL, SPPB, Rosow and Breslau disability scale, short form-12 questionnaire, Physical Function Questionnaire, and sarcopenia
Coelho-Junior, H.J., 2021, Europe [41]	Community-dwellers and institutionalized older adults, free of disability and dementia	Systematic review and meta-analysis	Med diet adherence association with incidence of mild cognitive impairment (MCI), dementia, and/or Alzheimer’s disease (AD), and/or changes in physical performance	19,734	68.8 to 86.3 years	Walking speed (SMD = 0.42; *p* = 0.006), knee muscle strength speed (SMD = 0.26; *p* < 0.00001)	Cognitive function, physical performance, and adherence to the Mediterranean diet
Dominguez, L.J., Spain, 2023 [12]	Community dwellers	Systematic review and meta-analysis	Examine the relationship between adherence to the Mediterranean diet and the risk of developing frailty in older adults	103,615	±75 years	Higher adherence to the Mediterranean diet was associated with a significantly lower risk of developing frailty. OR 0.55 (0.44–0.70), lower risk of frailty for higher adherence to the diet. *p* < 0.0007	Adherence to the Mediterranean diet

Mediterranean Diet Score (MDS), Food Frequency Questionnaire (FFQ), Activities of Daily Living Scale (ADLS), Instrumental Activities of Daily Living scale (IADL), Short Physical Performance Battery (SPPB), HGS (hand grip strength), PA (physical activity), BMI (Body Mass Index).

## Abbreviations

The following abbreviations are used in this manuscript:

MD	Mediterranean diet
MeDiet	Mediterranean diet
MDS	Mediterranean diet score
aMED	Alternate Mediterranean diet score
MEDAS	Mediterranean Diet Adherence Screener
FFQ	Food Frequency Questionnaire
KHEI	Korean Healthy Eating Index
DASH	Dietary Approaches to Stop Hypertension
EWGSOP	European Working Group on Sarcopenia in Older People
EWGSOP2	European Working Group on Sarcopenia in Older People 2
DEXA	Dual-energy X-ray absorptiometry
HGS	Hand grip strength
BBS	Berg Balance Scale
FSST	Four Square Step Test
SPPB	Short Physical Performance Battery
ADL	Activities of Daily Living
IADL	Instrumental Activities of Daily Living
IG	Intervention group
CG	Control group
OR	Odds ration
CI	Confidence interval
SGLT2	Sodium–glucose transport protein 2
CRT	Calorie restriction therapy
PA	Physical activity
BIA	Bioelectrical impedance analysis
BMI	Body Mass Index
